# True Tapping Mode Scanning Near-Field Optical Microscopy with Bent Glass Fiber Probes

**DOI:** 10.1155/2018/3249189

**Published:** 2018-04-22

**Authors:** A. Smirnov, V. M. Yasinskii, D. S. Filimonenko, E. Rostova, G. Dietler, S. K. Sekatskii

**Affiliations:** ^1^Laboratoire de Physique de la Matière Vivante, IPHYS, Ecole Polytechnique Fédérale de Lausanne, BSP-408, 1015 Lausanne, Switzerland; ^2^Institute of Physics, National Academy of Sciences of Belarus, Prospekt Nezavisimosti 68, 220072 Minsk, Belarus

## Abstract

In scanning near-field optical microscopy, the most popular probes are made of sharpened glass fiber attached to a quartz tuning fork (TF) and exploiting the shear force-based feedback. The use of tapping mode feedback could be preferable. Such an approach can be realized, for example, using bent fiber probes. Detailed analysis of fiber vibration modes shows that realization of truly tapping mode of the probe dithering requires an extreme caution. In case of using the second resonance mode, probes vibrate mostly in shear force mode unless the bending radius is rather small (ca. 0.3 mm) and the probe's tip is short. Otherwise, the shear force character of the dithering persists. Probes having these characteristics were prepared by irradiation of a tapered etched glass fiber with a CW CO_2_ laser. These probes were attached to the TF in double resonance conditions which enables achieving significant quality factor (4000–6000) of the TF + probe system (Cherkun et al., 2006). We also show that, to achieve a truly tapping character, dithering, short, and not exceeding 3 mm lengths of a freestanding part of bent fiber probe beam should also be used in the case of nonresonant excitation.

## 1. Introduction

Scanning near-field optical microscopy (SNOM) technique enables overcoming Abbe diffraction limit of far-field optics and obtaining simultaneously optical and topographical images; see, for example, [1–3] for recent reviews. While the optical resolution of the method is limited by an aperture size and is typically 50–100 nm, an excellent spatial resolution in a topography channel quite comparable with Atomic Force Microscopy and enabling in particular cases even the imaging of individual* single strand* DNA molecules [4] can be realized. Naturally, we need a convenient and precise method to control the distance between the tip and sample for the successful operation of any SNOM device.

The most popular method of the SNOM tip-sample distance control is the shear force-based feedback employing a fiber attached to the quartz tuning fork (TF) first introduced in 1995 [5]. In this method, tip oscillates almost parallel to the surface of the studied sample with a few nanometers amplitude. Scientific community much debated in the nineties and the beginning of XXI century [6–9] the origins and physical mechanisms of the shear force interaction. As an explanation, different concepts were proposed such as time-varying attractive Van der Waals and capillary forces acting on the tip [6] or actual contact between the fiber and a specimen. However, some related debates still sometimes take place. Nowadays, it seems established that a real contact between tip and sample surface occurs at a certain point [7–11]. In other words, there is no such a significant difference between shear force and tapping modes. However, when the former is operative, the oscillation occurs at somewhat unfavorable conditions with the angle between the velocity of the movement of the fiber probe tip and normal to the sample surface approaching ninety degrees. As a result, it appears that shear force distance control method is far from the ideal one. The crosstalk between optical and topographical image can warp the results. The forces between the tip and sample are high and easily might be destructive. In particular, the* direct* measurement of the interaction force in combined SNOM-AFM device gives the values around 100–200 nN [9].

These considerations lead many experimental groups to propose and implement a tapping mode SNOM feedback when the probe tip apparently moves roughly perpendicularly to the sample surface; see, for example, [12–18]. A few realizations of this approach were based on straight optical fibers or short fragments thereof correctly attached to the tuning fork or bimorph [15, 16]. However, the use of natural long but* bent* optical fibers attached to the tuning fork in a standard fashion seems was the most popular [17]. However, the detailed analysis of the relative fiber-sample surface motion in these experiments has not been performed. Herewith the “true tapping” character of this motion (comparable with the motion of AFM cantilever in the tapping mode regime, see the end of “Simulations”) was taken for granted.

In the current paper, we report the realization of a new approach to the problem: bent sharpened glass optical fibers with carefully controlled (and small) sizes of the bent part and the radius of the curvature of the bending were prepared and experimentally exploited as SNOM probes. The design of these probes has been based on detailed theoretical and numerical studies of the relative tip-sample surface motion. We showed that these same aforementioned small sizes are* necessary* to achieve the tapping mode; otherwise, the “shear force type” interaction not only persists but very often* dominates* the whole picture. In our opinion, there are substantial grounds to suppose that the most of the earlier reported tapping mode SNOMs were* not* the devices exploiting the tapping mode.

## 2. Simulations

The commercially available software package ANSYS 17.2 (Canonsburg, PA, USA) was used for the numerical simulations. These simulations are based on the Finite-Element method (FEM) [19] exploiting the triangulated models of the tip (“meshing”) created via commercially available software package for modeling SolidWorks 2016 (Waltham, MS, USA). The local mesh size of the structure was based on the local curvature to cover all important (and possibly tiny) features of its design. The probe beams were hinged at the base. As a material, pure silicon was chosen for the AFM cantilevers and quartz for the fiber probes. The change of material properties (such as density *ρ*, elastic modulus *E*, and Poisson ratio) does not impact the mode shape but, of course, strongly influences the absolute values of resonant dithering frequencies, see below.

**Figure 1:**
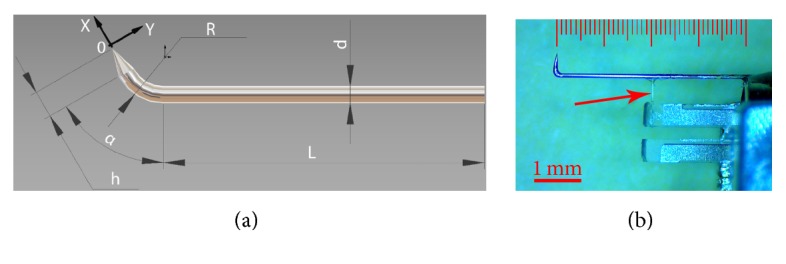
Illustrating the geometry of the bent fiber. (a) The following sizes of the bent fiber probe are shown: *α*, bending angle; *R*, bending radius; *d*, fiber diameter; *L*, length of the fiber from the point at which it is rigidly fixed until the bent sector; *h*, length of the probe's free part. (b) You can see the photo of a real bent fiber attached to the TF; here *α* ≈ 90°, *R* ≈ 300 *μ*m, *d* = 125 *μ*m, *L* = 4 mm, and *h* ≈ 300 *μ*m. Thin glass fiber-made driving rod connecting the probe with one of the TF's prongs is shown with a red arrow.

We introduce the following notation (see Figure 1). The local symmetry axis of the probe's tip, see Figure 1, is taken as *Ox* while the axis perpendicular to *Ox* and lying in the same plane as the *Ox* axis and the symmetry axis of the unbent part of the probe is taken as *Oy*. (The third axis, *Oz*, is not important since lateral oscillations in this direction are negligible.) The origin of the coordinates was put at the point coinciding with the tip apex. For probe vibrations at the frequency *ω*, the motion of the tip apex can be expressed as $\vec{r}\left(t\right)=A_{x}\vec{i}\cos\left(\omega_{x}t+\varphi_{x}\right)+A_{y}\vec{j}\cos\left(\omega_{y}t+\varphi_{y}\right)$, where at a moment we are not interested in the phases *φ*_*x*,*y*_. We normalize the ratio between the amplitudes of the oscillations in *Ox* and *Oy* directions as

(1) $$\begin{array}{c} \psi =\frac{A_{x}^{2}}{A_{x}^{2}{+A}_{y}^{2}} \end{array}$$

and name this quantity depending only on the probe characteristics and ranging between 0 and 1 “the tapping mode efficiency”: if the sample surface is parallel to the *Oy* axis, it gives the ratio of “tapping mode type” and “shear force type” movements of the probe apex.

For straight, unbent glass fiber without a tapered part (i.e., ordinary cylinder), the resonant dithering frequencies can be found from the following equation (see, e.g., [20]):

(2) $$\begin{array}{c} f_{res,n}=\frac{\Omega_{n}^{2}}{L^{2}}\sqrt{\frac{EI}{\rho S}}=\frac{\Omega_{n}^{2}r}{4\pi L^{2}}\sqrt{\frac{E}{\rho }}. \end{array}$$

Here *Ω*_*n*_, *n* = 1, 2, 3,…, are ordered *Ω*_*j*_ > *Ω*_*i*_ if *j* > *i* solutions of the characteristic equation cos⁡*Ω*_*n*_cosh⁡*Ω*_*n*_ + 1 = 0, and we have taken into account the dependencies of the cylinder beam cross section *S* and its inertia moment *I* on the radius *r* = *d*/2 = 62.5 microns. Using the known values *Ω*_1_ = 1.88, *Ω*_2_ = 4.69, *Ω*_3_ = 7.13 [21], we see that the probe resonantly oscillates at the TF working frequency 32,768 Hz if the lengths of the freestanding part of the probe are equal to 1.7, 4.3, and 6.6 mm, respectively, for the first, second, and third resonances. For our proprietary, so-called “double-resonant” montage of the SNOM probe onto TF, the exact coincidence of the working frequency of the latter with the second resonant frequency of the fiber probe beam is realized [22, 23]. In the same works, we have shown that this enables achieving very large quality factor of the “TF + fiber probe glued onto it” system. Thus it leads to the small acting forces and giving possibilities to excellent spatial resolution in a topography channel.

**Figure 2:**
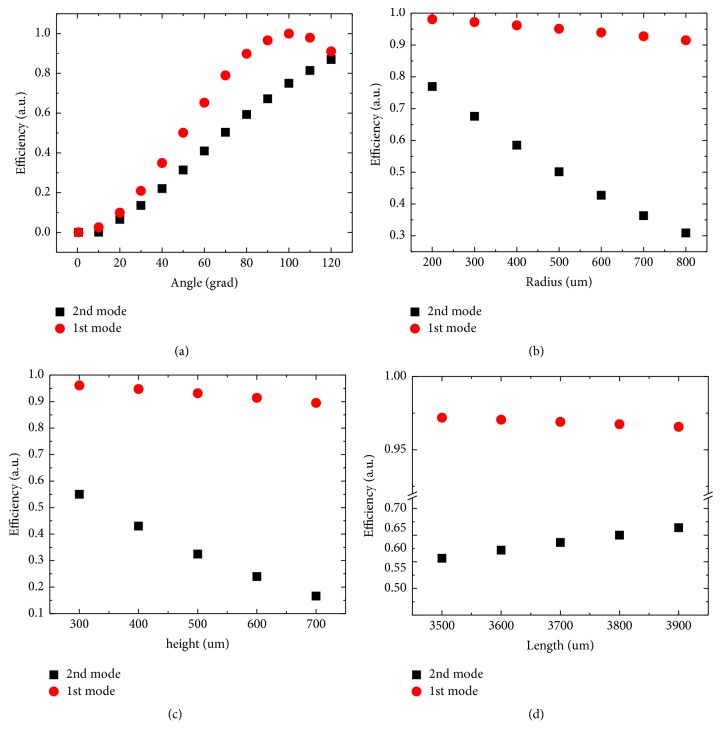
Simulation results for the 1st and 2nd resonance mode of the fiber with 125 *μ*m diameter. (a) Efficiency on the bending angle dependence *ψ*(*α*). *R* = 300 *μ*m; *L* = 4 mm; *h* = 200 *μ*m. (b) Efficiency on the bending radius dependence *ψ*(*R*). *α* = 90°; *L* = 4 mm; *h* = 200 *μ*m. (c) Efficiency on the free part length dependence *ψ*(*h*). *α* = 90°; *R* = 300 *μ*m; *L* = 4 mm. (d) Efficiency on the fiber length dependence *ψ*(*L*). *α* = 90°; *R* = 300 *μ*m; *h* = 200 *μ*m.

In Figure 2, we present the simulation results pertinent to the first and second resonance modes of the fiber probe dithering. These graphs express an idea about the level of impact of different parameters on the tapping mode efficiency (*ψ* was calculated from amplitudes of the oscillations in *Ox* and *Oy* for probes with different parameters according to (1)). The exact values of the resonant frequencies slightly depend on the parameters of the probe being close to the values of 5,140 Hz and 32,768 Hz, respectively, for the first and second resonances.

The main conclusion following from this simulation is the clear difference between the first and second resonances. While for the first resonance mode and the probe bending angle of 90°, the tapping mode character of the probe's tip motion does take place for a rather broad range of the probe parameters (as it seems intuitively evident). To achieve high tapping mode efficiency for the usually used (see Figure 2) second resonance mode, careful control of the probe's free part length *h* and its curvature radius *R* are needed. Otherwise, the shear force type motion dominates (which looks somewhat counterintuitive). Both these parameters should be as small as possible. The length of the probe *L* does not strongly affect this same efficiency. However, the varying of this length can be used to adjust the resonance frequency of the probe without seriously influencing its other characteristics. The exploitation of the* third* resonance mode gives the quite poor quality of the tapping mode (data are not shown in Figure 2), so it does not seem attractive to work in such configuration.

Indeed, the minimization of both *h* and *R* parameters is limited by the very diameter *d* = 125 micron of the standard glass optical fiber used to prepare SNOM probes. Moreover, such a minimization presents technical challenges already when the values are two-three times bigger than *d*. We succeeded to solve these challenges (see Section 3), and in Figure 1(b) we present an optical image of one of the fiber probes used in the SNOM experiments reported below. Note that for our experiments we were using 2nd resonance mode.

For the “state-of-the-art” fiber probe (*h* and *R* values are as small as possible, namely, *α* = 90°, *R* = 250 *μ*m and *h* = 300 *μ*m), we calculated the tapping mode efficiency of the second resonance mode according to (1). The computed efficiency is approximately *ψ* = 0.6: a good value certainly exceeding one half, which enables us to reasonably speak about the tapping mode for the case. But it is still far from the unity. As attested by the Figures presented, simulations show that even not so large increase of the aforementioned crucial probe parameters rapidly results in the values of *ψ* well below 0.5. That is to large extent makes the simple* mentioning* of the tapping mode character of the probe motion meaningless. It can be said that, up to now, this is exactly the quite typical situation in the field. Again, the exploitation of common nonresonance (i.e., characterized by an arbitrary and not well controlled length of the freestanding part) SNOM probe (with classical TF) cannot drastically change the situation with the tapping mode efficiency. It arises from the origins of nonresonance mode, a combination of a few resonances with the main contribution from the second one.

The actual motion of the fiber probes when second resonance dithering mode is operative, illustrating both mostly “shear force” and “tapping mode” behavior as a function of the probes' sizes can be seen in Supplementary Materials (available here).

**Figure 3:**
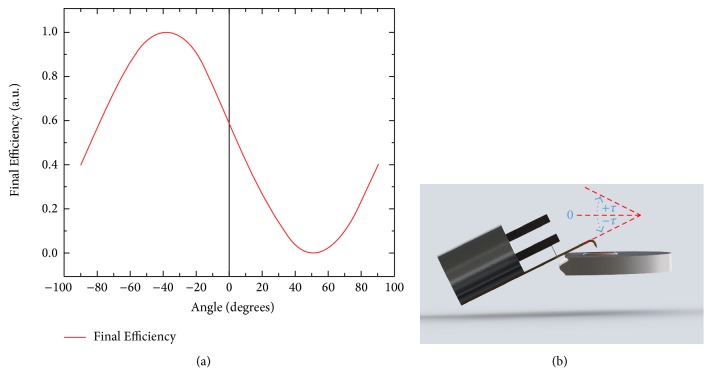
Rotation of the tip (initial *ψ* = 0.6) results in the change of the *ψ* factor. (a) It can be seen the geometry of problem: bent fiber attached to the TF in double resonance condition is tilted relative to the sample. Rotation angle *τ* is shown in (b). One can see that the rotation in the negative direction is limited by the very sample.

Indeed, by varying an angle *τ* between the sample surface and *Ox*, *Oy* axis (Figure 3), the ratio of the “tapping type” and “shear force type” motions can be changed following the simple geometrical formula, which arises from the rotation matrix:

(3) $$\begin{array}{c} \psi^{\prime }=\left(\;1-\psi \;\right){\left(\;\sin\left(\;\tau \;\right)-\sqrt{\frac{\psi }{1-\psi }}\cos\left(\;\tau \;\right)\;\right)}^{2}. \end{array}$$

We illustrate this circumstance on Figure 3(a) by presenting the data pertinent for our optimized fiber probe with the parameters above *α* = 90°, *R* = 250 *μ*m, and *h* = 300 *μ*m (*ψ* = 0.60). Note, however, that the apparent increase of the tapping mode efficiency for negative rotation angles very often is only an illusory one and cannot be realized in practice due to geometrical limitations, see Figure 3(b).

**Figure 4:**
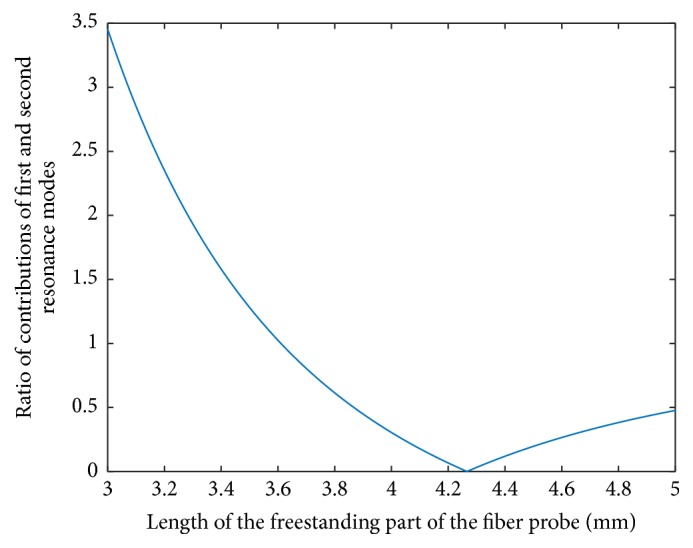
The ratio of the contributions of the first and second resonance vibration modes of the fiber probe beam (factor |*ω*^2^ − *ω*_2_^2^|/|*ω*^2^ − *ω*_1_^2^|) to the amplitude of the dithering of the probe as a function of the length of the freestanding part of the beam when excited at 32,768 Hz.

Let us now briefly analyze what is the situation with the tapping and shear force dithering modes when a “more standard” gluing of the fiber directly on one of the TF's prongs implying the subsequent nonresonant excitation takes place. Such approach does not preserve initially high-quality factor of the TF electromechanical oscillations, its value diminishes typically to around 300–500 and often even lower, correspondingly leading to essentially larger acting forces. It is well known that at conditions of nonresonant excitation of a beam at a frequency *ω*, the shape of the dithered beam (“deflection curve”) is given by the weighted sum of the shapes of the corresponding *n*th normal modes of vibration of the beam. In our case, these are the modes pertinent to the dithering of a rod whose one end is hinged and another free. The relative weights of the corresponding contributions are proportional to |*ω*^2^ − *ω*_*n*_^2^|^−1^; see, for example, [21]. Indeed, any system having distributed mass and elasticity can be described in this way. Using formula (2) with the known ratio *ω*_2_/*ω*_1_ = *Ω*_2_^2^/*Ω*_1_^2^ = 6.22, we immediately see that a severe caution should be paid to the length *L* of the fiber beam if one wants to realize a tapping mode probe-sample interaction. This situation is illustrated in Figure 4: for the values of *L* smaller than *L* = 3 mm, the contribution of the first resonance mode dominates. However, already for this length, the second resonance contributes already roughly one-third of the total amplitude of a dithering. The contribution of the first resonance mode rapidly becomes negligible for larger values of *L*. Taking into account the aforementioned performance of the second dithering mode, it can be said that often the claimed tapping mode character of the bent fiber probe dithering is not at all such one. In real, the usability of the probe with the length *L* less than 3 mm is severely limited because of the difficulties to work with short probes. In such a way the particular design of the probe should be used.

**Figure 5:**
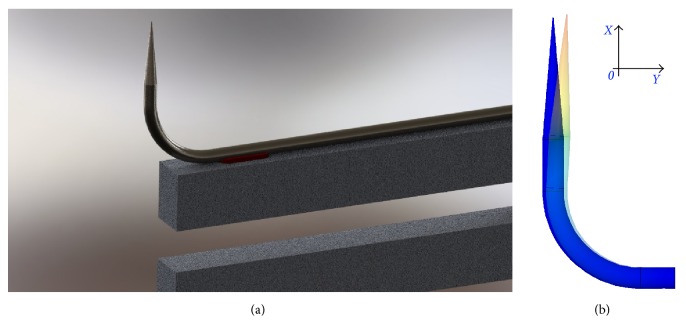
Illustrating the typical probe using nonresonant excitation scheme. (a) The model of such a scheme, here *α* ≈ 90°, *R* ≈ 500 *μ*m, *d* = 125 *μ*m, *L* = 200 *μ*m, and *h* = 800 *μ*m. (b) Dominant oscillation mode 36 kHz, *ψ* < 0.05.

At the same time, one should keep in mind that with decreasing of values of the length *L* and keeping the relatively long probe's free part *h* new resonance mode will come into play. We studied the most popular scheme of the bent fiber attached to the TF in nonresonant conditions (see Figure 5(a), similar parameters of bending were used in [17]). Such a rush method of fiber bending and attaching to the TF can lead to the reduced tapping mode efficiency due to the mode excitation on the probe's free part (see Figure 5(b)), whereas oscillations happen mostly in the *Oy* direction; the value of *ψ* for this mode is less than 0.05. Additionally, the longer the probe's free part is, the worse the tapping mode efficiency is. For disturbing modes elimination, short fiber tips should be used; namely, *R* and *h* values have to be rather small.

Finally, let us note that similar numerical analysis of the standard* AFM probes* (a cantilever 0.5–5-micron thick, 50–200-micron long, and 20–50 micron in width rectangular beams with a 10–30-micron long pyramid tip “elastically” attached to them is taken as a standard; the whole probe is considered as made from one initially intact piece of material without any gluing) reveals a truly tapping mode character of interaction there: the factor *ψ* ranges 0.95 and more for the case for the first resonance. At the same time, for the most, AFM setups cantilever tilting of 10–15 degrees concerning the normal to the sample surface leads to the drop of the factor *ψ* down to ca. 0.75 making it comparable with the optimized bent SNOM probes discussed below and working in the second resonance mode.

## 3. Experiments

Bent optical fiber-made SNOM probes were prepared as follows. Single-mode glass optical fibers FS-SN-3224 from 3 M (Maplewood, MN, United States) with 125 *μ*m diameter were dipped into a ca. 40% HF water solution with vacuum oil overlayer without stripping the polymer coating (so-called tube etching method [24]) and etched for 120 minutes. After the etching, the polymer coating was dissolved in hot concentrated H_2_SO_4_. The temperature of HF solution was carefully controlled and was maintained at 35 ± 0,05°С.

The bending of the sharpened fibers occurs under the effect of their focused CO_2_ laser irradiation that locally heats the quartz nearby the tip up to the melting point. The tip of the fiber is pushed towards the laser beam due to the surface tension forces arising in the area facing the beam where quartz melts faster than on the opposite side. By changing the power of the incident laser radiation and the size of the focal spot, it is possible to control the bending radius and angle.

**Figure 6:**
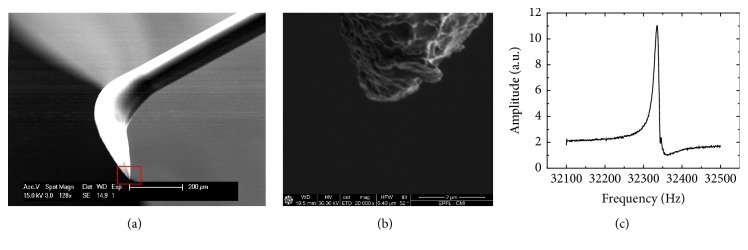
(a) SEM image of the Al-coated bent fiber tip. (b) SEM image of the tip apex marked with a red frame on the picture (a). (c) Amplitude frequency characteristics of the bent fiber probe attached to the TF.

Next stage of the probe preparation consists in the fabrication of the subwavelength-size aperture for the light transmission onto their apex. Different attempts to realize a known shadow coating procedure [25, 26] for this purpose were undertaken, but they all failed due to the much more complex geometry of our probe in comparison with the straight one. Therefore, the blind metal coating was used together with the subsequent opening of the subwavelength aperture using Focused Ion Beam milling technique; see Figure 6(a) for the SEM image of the metal-coated fiber and Figure 6(b) for the close-up of the very apex of the coated fiber. For the coating, an aluminum layer with ~150 nm thickness was deposited exploiting Alliance-Concept EVA 760 e-beam evaporator. In a few cases, the preparation of the subwavelength aperture by simple intensive scratching of the initially blind entirely metal-coated probe over an appropriate sufficiently “rigid” sample (e.g., a glass slide) also has been used.

The finished bent SNOM probes were glued onto the quartz tuning fork in the double-resonant conditions following the procedure outlined in [23]. Since such montage requires the matching of the frequencies, it is essential to glue carefully both the fiber onto the metal case of the TF (to create the “hinging point”) and 40-micron diameter glass driving rod connecting the fiber and one of the TF's prongs (see Figure 1) and precisely control the distance *L*. It was found that this distance should be equal to 3.8 ± 0.1 mm, in the case of the use of 125 *μ*m FS-SN-3224 fiber-made probe with the following bending parameters: *α* = 90°, *R* = 250 *μ*m, and *h* = 300 *μ*m. The driving rod connecting the probe with one of the TF's prongs should be glued at ca. 2 mm distance from the fiber hinging point. Such a method of attaching of fiber to the tuning fork results in quite large quality factor: initial value of *Q* lying in the range of 10,000–12,000, which is characteristic for a free unloaded tuning fork in air, drops down to the values ranging 4000–6000 after proper gluing of a glass fiber probe onto it. For illustration, in Figure 6, we present a typical Amplitude Frequency Characteristic of the bent fiber attached to the TF in double resonance conditions (the *Q*-factor is approximately 4300) together with the SEM images of the probe tip.

**Figure 7:**
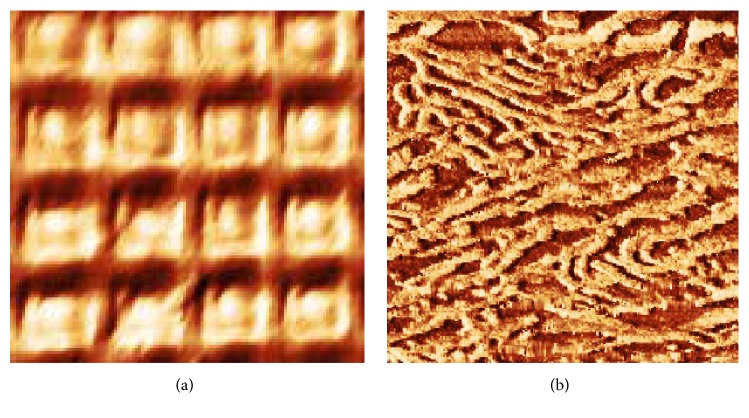
(a) The topography of the calibrating grating 2000 lines/mm, 2.0 *μ*m × 2.0 *μ*m. (b) The topography of the close-packed Phi X 174 DNA, 0.7 *μ*m × 0.7 *μ*m. The images were obtained in constant *Q*-factor mode.

The performance of the aperture bent SNOM probes has been assessed exploiting them as probes of the slightly modernized customarily made scanning near-field optical microscope, whose main features were discussed earlier [4, 23]. In Figure 7(a), we present the topographical image of 677-AFM calibrating grating, 2000 lines/mm (Ted Pella, Redding, CA, USA) routinely used to calibrate Atomic Force Microscope. As a more severe test, mica samples containing densely deposited DNA molecules onto APTES-modified surface, see, for example, [27], for preparation details and AFM images, were used; see Figure 7(b).

**Figure 8:**
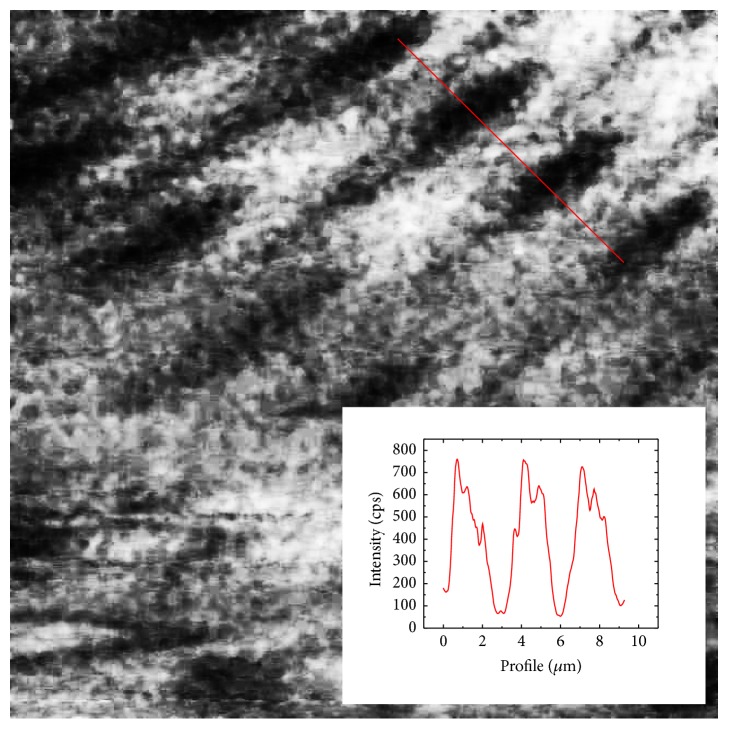
The optical image (20 *μ*m × 20 *μ*m) of the SNG01 SNOM calibrating grating-rhomb vanadium islands 20–30 nm in thick on the quartz substrate (NT-MDT, Zelenograd, Russia) obtained in transmission mode; cross section along the red line is shown on the bottom.

The near-field optical image obtained exploiting these tapping mode bent probes is presented in Figure 8 (UV image of such sample can be found in [28]).

## 4. Results and Conclusion

We have presented the truly tapping mode scanning near-field optical microscopy with single-mode glass optical fiber-made bent probes. Extensive numerical simulations enabled clarifying the conditions necessary to achieve a tapping rather than shear force probe-sample interaction (and also to quantify this initially not a clear-cut concept). Based on these simulations, we prepared such probes and tested them in the real SNOM device; not only calibration gratings but also mica-deposited DNA molecules were successfully imaged. Tapping mode efficiency of our probes, working in the conditions of second resonance dithering mode, lies in the range 0.6–0.7 which is not too far from the AFM cantilevers efficiency in the case of a standard setting. It is essential to mention that bent optical fiber-based SNOM probes prepared without taking into account the simulation above results and following from them understanding indeed often cannot be considered as working in a tapping mode.

It was shown [29] that tapping mode SNOM is preferable in studies of soft biological samples. Furthermore, it can operate in liquids with much better performance [18]. We mentioned setups with the vertical oscillation of the tip which can be considered as a true tapping without doubts. Meantime, we demonstrate how to realize tapping mode SNOM using bent fibers. Due to the use of our proprietary double-resonant montage of these bent probes onto the tuning fork [22, 23], similarly small acting interaction forces, lying in a few nano- or even sub-nano-Newton range, are achieved. This paves the way to use them for the imaging of fragile biological samples and future work in liquid.

## Supplementary Materials

Supplementary Materials R100.avi – Actual motion of the fiber probe in the second resonant mode with the bending radius *R* = 100 *μ*m and therefore the motion of the tip can be described as mostly “tapping mode”. R800.avi - Actual motion of the fiber probe in the second resonant mode with the bending radius *R* = 800 *μ*m and therefore the motion of the tip can be described as mostly “shear force”.
