# Supplementary material for: True Tapping Mode Scanning Near-Field Optical Microscopy with Bent Glass Fiber Probes
Source: Scanning. 2018 Apr 22;2018:3249189. doi: 10.1155/2018/3249189 (PMC5937387; doi:10.1155/2018/3249189)
Supplement: Supplementary Materials — R100.avi – Actual motion of the fiber probe in the second resonant mode with the bending radius R = 100 μm and therefore the motion of the tip can be described as mostly “tapping mode”. R800.avi - Actual motion of the fiber probe in the second resonant mode with the bending radius R = 800 μm and therefore the motion of the tip can be described as mostly “shear force”. [file 3249189.f1.zip › 3249189.f1/3249189_SupplDesc.docx]

# Supplementary materials

R100.avi – Actual motion of the fiber probe in the second resonant mode with the bending radius $R=100 \mu m$ and therefore the motion of the tip can be described as mostly “tapping mode”.

R800.avi - Actual motion of the fiber probe in the second resonant mode with the bending radius $R=800 \mu m$ and therefore the motion of the tip can be described as mostly “shear force”.
